# Nursing Care for Patients Treated with Chemotherapy for Bone Tumours

**DOI:** 10.1080/135771402753667718

**Published:** 2002-05


### NURSING CARE FOR PATIENTS TREATED WITH CHEMOTHERAPY FOR BONE TUMOURS

### ORAL PRESENTATIONS

### H130 A Structured and Effective way to Inform Sarcoma Patients

N.A.C. Leyerzapf, C. Mewe
*Dept. Of Orthopaedic Surgery, Leiden University Medical Center, Leiden, The Netherlands*

The Orthopaedic department, in the University Medical Centre, is one of the main centres for the treatment of bone tumours in the Netherlands. Each year about 1000 operations are carried out on this department, approximately 200 of these are on patients with a bone tumour (benign as well malignant). The patient category is usually young and in an important period of their life (school/study, sport, relationships, children, etc). The investigations, diagnosis and treatment often have a (adverse) psychological/social effect on the patient and his/her environment. A mutilating operation often results in an identity crisis. During the time in the hospital, the patient comes into contact with various specialists (of all levels), this results in receiving a great deal of information in a short matter of time. We developed information folders and video's for the patients to have more clearness about what will happen to them. This process of development is still going on. It's also possible to have contact with fellow cancer patients before or after operation. By using a protocol and an information checklist we reduce the risk that the patient has not understood certain items or received conflicting information concerning the plan of treatment. Two days before discharge, the nurse assigned to the patient will go through a checklist with him/her. This is to be certain that everything is clear before discharge, to evaluate if the nursing and doctors care, or other relevant factors were satisfactory, and suggestions for improvements in the future.

### H131 Nursing Care for Patients Treated with Chemotherapy for Bone Tumours the 'Euro-Ewing 99' Protocol

E.D. Ouwerkerk
*Leiden University Medical Center, Leiden, The Netherlands*

Since December 1999 patients in the Leiden University Medical Center are being treated with the so called 'Euro-Ewing 99' protocol. Before this protocol the patients were treated with 14 cycles of chemotherapy with or without radiation therapy. The new protocols main objective is to enlarge the 5 years survival rate. In the new protocol all patients receive 6 cycles of chemotherapy, (induction treatment) with no restrictions for stage of the disease. The cycles are given once every three weeks. After these first 6 cycles the patients undergo orthopaedic surgery also in our hospital. After the surgery the treatment choice is to be determined on according to the response of the tumour to the chemotherapy. The follow-up treatment consists of another 8 cycles chemotherapy with or without radiation or high dose chemotherapy with peripheral stemcel transplantation. Patients often hear their diagnosis on the outpatient clinic from their orthopaedic surgeon or from their oncologist. The oncologist will inform the patients about their oncoming treatment and the side effects (informed consent) At our department of clinical oncology the patients receive the chemotherapy for which they are admitted to the hospital for four days. The category of patients are mainly adolescents and young adults. The nurse will inform the patient once more about the oncoming treatment and the side effects. They also receive written information directly related to the treatment. Five patients were treated on our ward according to this new protocol. One patient said, the treatment was physically as well as psycho-social very intensive.

### H132 How to Inform the New Sarcoma Patient?

C. Forni, L. Loro, T. Mazzei, C. Beghelli, M. Tremosini, A. Biolchini, A. Triggiani, C. Raspanti
*Istituti Ortopedici Rizzoli, Bologna, Italy*

When a new patient comes to a Chemotherapy ward, there is a lot of information he/she needs to know in a very short time. Nurses play a key role in this field. During the last ten years the nursing team of the Chemotherapy Service at Rizzoli Institute have written various educational booklets on this topic. We started with producing a patient's booklet on Chemotherapy and the main role and organization of our ward to be given at each new patient during the first admission in our ward. After several months we carried out a survey amongst patients at the end of their treatment. We wanted to check whether the information given at the beginning of their treatment had been of any use. The survey results gave us useful suggestions for our further information booklets and leaflets:

- What Chemotherapy is and the role of nurses, doctors and patients;
- Specific information on drugs used: Methotrexate, Vincristine, Ifosfamide, Cisplatin and Adriamicina;
- How the school service work in our word;
- What GCSF is, the practical procedures of using them and their side effect;
- The differences between Peripheral and Central Venous Catheter;
- The differences between intravenous and intrarterial therapy:

The up dating of our booklets and leaflets is an ongoing process. In this paper we would like to discuss and compare methodologies and materials with colleagues who work in the same field.

### H133 Wound Healing Deficiencies after Major Tumour Operations

Lübben, K.
*Uniklinikum Münster, Orthopädie Station, Münster, Germany*

Wound healing deficiencies after tumour operations especially by patients who are immune deficient and also because of a reduction of the bed facilities, are often seen in our daily practice. Due to this we now have experience with the principle of treating these wounds. We use the procedure of 'wet wound treatment'. During our presentation we would like to introduce our patients, their surgical procedure and our daily practice with the specific wound treatment. We will present the principle, the products and how to put it into daily practice. We will make use of documented photos of wound deficiencies seen in some of our patients and the progress made by the treatment throughout the healing process.

